# Supplementary material for: Decreasing extents of Archean serpentinization contributed to the rise of an oxidized atmosphere
Source: Nat Commun. 2021 Dec 20;12:7341. doi: 10.1038/s41467-021-27589-7 (PMC8688491; doi:10.1038/s41467-021-27589-7)
Supplement: Supplementary file 1 — Supplementary Information [file 41467_2021_27589_MOESM1_ESM.pdf]

SUPPLEMENTARY MATERIAL

DECREASING EXTENTS OF ARCHEAN SERPENTINIZATION CONTRIBUTED TO THE RISE OF AN OXYGENIC ATMOSPHERE

James Andrew M. Leong[1*#], Tucker Ely[1##], and Everett L. Shock[1,2]

[1]School of Earth and Space Exploration, Arizona State University, Tempe, AZ, USA

[2]School of Molecular Sciences, Arizona State University, Tempe, AZ, USA

*corresponding author: jmleong@asu.edu

current address: [#]Lamont-Doherty Earth Observatory, Columbia University, New York NY, USA

[##]Department of Earth and Environmental Sciences, University of Minnesota, Minneapolis, MN, USA

Supplementary Note 1

H_2-generation potentials of various Fe-bearing igneous rocks

The amount of H_2 predicted to form during low-temperature (25 °C) alteration of various Fe-bearing igneous rocks is summarized in Figures 1 and 2. Connections between H_2-generation potentials, MgO content, and abundance of other major oxide components of the rocks are shown in Figure S1. A total of 9,414 different Fe-bearing igneous rocks of variable compositions were used for the rock alteration models. Compositions of reacting rocks were taken from a precompiled list of Fe-bearing igneous rocks (komatiite, picrite, peridotite, harzburgite, dunite) in the GEOROC database. Only samples with complete major oxide data were used. Furthermore, only major oxide data for MgO, SiO_2, FeO, CaO, Na_2O, and K_2O,

normalized to 100% on an anhydrous basis, were used in the calculations. Most of major oxide data were determined on whole rock materials using either x-ray fluorescence or mass spectroscopy. Almost all of the samples in the precompiled lists report the total FeO. Otherwise, total FeO was calculated if both Fe(II)O and $Fe(III)_2O_3$ were indicated (total FeO = Fe(II)O + $0.9Fe(III)_2O_3$). Calculations and plots shown in Figure S1 use total FeO values. As shown in Figure S1a, the total FeO contents of the vast majority of rocks used in the simulations are closely similar (6–14 wt% FeO) and exhibit a weak trend with the MgO content. The potentials to generate H_2 during alteration of these rocks vary, despite having similar amounts of ferrous iron that can potentially be oxidized and yield H_2 from the coupled reduction of H_2O. Instead of the FeO content of the rocks, their potential to produce H_2 is tied to their bulk compositions, which in turn dictate how Fe is distributed and speciated in the secondary assemblages during rock alteration. Generally, alteration of MgO-rich rocks (>35 wt% MgO) results in the highest potential to generate H_2 (Figures 1a and 1b), except for some that are the most enriched in MgO (>45 wt% MgO). MgO-rich rocks tend to be depleted in SiO_2, CaO, Al_2O_3, Na_2O and K_2O. Hence, as shown in Figures S1b to S1f, the potentials to generate H_2 during rock alteration tend to decrease, depicted by the transition to cooler colors in Figure S1, as the reacting rocks become more enriched in these chemical components.

Overall, the potential to generate H_2 closely trends with the amount of serpentine that forms in the models, as shown in Figures 1c and 1d. Precipitation of brucite, chlorite, talc, and clay minerals can sequester large proportions of unoxidized ferrous iron into their crystal structures and inhibit H_2 production

through reaction (2). In contrast, serpentine minerals partially exclude ferrous iron from their crystal structures and accommodate ferric iron instead. Several studies have shown that Fe(III)/ΣFe in serpentine can attain values above 0.5, with some observations indicating values up to 1[1,2], while chlorites typically have Fe(III) /ΣFe values of less than 0.1[3]. The mechanism explaining why serpentine excludes ferrous iron from its crystal structure relative to other minerals such as chlorite is yet to be fully explained. However, the partitioning of Fe(II) and Fe(III) into minerals could be imposed by several crystallographic and chemical variables such as the charge and size of substituting cations, the presence of other trivalent cations, and the need for coupled substitutions in host minerals[3].

As shown in Figures 1a and 1b, the potential to generate H_2 during alteration increases as the MgO content of the rock decreases from 50 to 40 wt% where serpentine is increasingly favored to form at the expense of brucite, which only hosts ferrous iron. In contrast, potentials to form H_2 decrease as the MgO content of the reacting rock decreases from 40 to 20 wt% as serpentine becomes less favored relative to chlorite. Moreover, as the SiO_2 contents of rocks typically increase with decreasing MgO content (see Figure S1b), the composition of the precipitating Fe-bearing serpentine trends toward the relatively more Si-rich Fe(II)-endmember (greenalite, $Fe(II)_3Si_2O_5(OH)_4$) instead of the ferric-iron bearing endmember (cronstedtite, $(Fe(II)_2Fe(III))(Fe(III)Si)O_5(OH)_4$), resulting in less H_2 formation[4,5]. Serpentine is not favored to form during the alteration of rocks with MgO content of less than 20 wt%, resulting in minimal potentials to generate H_2 during the alteration of these rocks (left side of Figures 1a and 1b). This range in rock composition

encompasses basaltic and picritic rocks where the dominant Fe-bearing secondary assemblages include chlorite, talc, and clay minerals (Figure 1c). Other minerals (gray symbols in Figure 1c) calculated to form in alteration simulations of basaltic and picritic rocks are predominantly zeolites, prehnite, secondary plagioclase, and tremolitic amphibole. Overall, serpentine and chlorite as the predominant secondary minerals for rocks with MgO content between 18 to 40 wt% (*i.e.*, compositional range of komatiites) is consistent with those observed from extant altered komatiites[6]. However, both serpentine and chlorite are stable at a wide range of temperature and pressure conditions[7,8] and observed mineral paragenesis could record several stages of alteration processes. Isotopic studies[9,10] show that some present-day serpentinites in ophiolites form through interactions with meteoric-derived groundwater under ambient conditions, consistent with equilibrium predictions. Isotopic evidence for komatiites altered at low temperatures is less known. In addition, lower temperature interactions may overprint previous higher temperature products[11]. Alternatively, attainment of equilibrium even at low-temperature conditions is supported by studies on the compositions of low-temperature (<40 °C) hyperalkaline and reduced fluids seeping out from ultramafic outcrops in ophiolites, which are present-day analogs of ultramafic-hosted groundwater during the Archean. Recent work[12] shows that while most hyperalkaline fluids are modified by shallow subsurficial or surficial processes, some of the most end-member-like fluids (highest pH, lowest dissolved Mg and Si concentrations) are consistent with equilibrium expectations. The formation of reduced and hyperalkaline fluids during serpentinization can involve several

thousands of years[13], which can ensure the approach to equilibrium even at low temperature conditions.

The highly variable H_2-generation potentials of rocks with MgO content between 30 to 45 wt%, as summarized in Figure 2, are due to the variable potentials to precipitate Fe(III)-bearing phases among rocks within this compositional range. The extents of these variations are shown in further detail in Figure S2 (groups 2, 3, and 4). A semi-logarithmic version of Figure 1a is shown in Figure S2a in which the black dots correspond to the grey dots in Figure 1a. The distribution of the H_2-generation potentials at compositional groups defined by the MgO content of the rocks is shown by the black curves in Figure S2b. Specifically, the black curves in Figure S2b depict distribution of H_2-generation potentials of rocks with MgO content >45 (group 1) and <10 wt% (group 9) as well as those from 45 to 10 wt% at 5 wt% intervals (groups 2 to 8). Other features of these plots are discussed below. As shown in Figure S1b, rocks with similar MgO content can have variable SiO_2 content. In most cases, those with higher SiO_2 content favor precipitation of the greenalite end-member of serpentine over cronstedtite. As more Fe(II) from primary minerals is transferred unoxidized to greenalite, less H_2 is generated. Lower H_2-generation potentials for rocks with similar MgO content but higher SiO_2 content are shown in Figure S1b (cooler colors). Overall, cronstedtite trends shown in Figure 1d are reflected in the H_2-generation potentials shown in Figures 1a and 1b. The varying potential to precipitate cronstedtite and greenalite in serpentine solid solutions is the primary reason why the distribution of H_2-generation potentials for rocks with MgO content between 35 and 40 wt% (black curve for group 3 in Figure S2b) appears

bimodal. In addition, a minor peak corresponding to lower H_2-generation potentials for rocks with MgO content between 40 and 45 wt% (black curve for group 2 in Figure S2b) corresponds to those rocks with higher SiO_2 contents (see Figure S1b). In contrast, minor peaks corresponding to higher H_2-generation potentials relative to most others for rocks with MgO content between 30 and 35 wt% (black curve group 4 in Figure S2b) correspond to samples with lower SiO_2 contents (see Figure S1b). While the alteration of the majority of rocks within this compositional group (group 4 in Figure S2b) yields similar H_2-generation potentials to groups with lower MgO contents, those yielding high H_2 contribute to increased likelihoods in offsetting significant amounts of O_2 production (see Figure 3 and 4) compared with results for groups with lower MgO contents.

In addition to serpentine, garnet, specifically that which is andraditic ($Ca_3Fe(III)_2Si_3O_{12}$) in composition, is another Fe(III)-bearing silicate mineral that can precipitate during low-temperature alteration. The precipitation of andradite garnet, and its hydrated equivalents (hydrogarnets), is invoked as a source for H_2 during serpentinization of peridotite bodies[14]. As shown in Figures 1c and 1d, garnet precipitation is favored as the MgO content of reacting rocks decreases from 50 to 35 wt% and contributes, in addition to serpentine, to the increasing potentials to generate H_2 (Figure 1a). Furthermore, rocks with similar MgO content can have variable CaO content and thus have varying potentials to precipitate andradite (see red symbols in Figures 1b and 1d) and further contribute to the highly variable nature of the H_2-generation potential of various compositional groups depicted in Figure S2. Overall, the alteration of rocks with MgO content <25 wt% yields lower

potentials to generate H_2 than those with higher MgO content. For these relatively Mg-poor rocks, there are two distinct H_2-generation trends as shown by the generally bimodal distribution of the black symbols and curves in Figures S2a and S2b (groups 6 to 9), respectively. One trend depicts higher H_2-generation potentials (10^{-4} to 10^{-2} Tmole H_2 (km^3 rock)$^{-1}$) while the other yields lower potentials (10^{-8} to 10^{-6} Tmole H_2 (km^3 rock)$^{-1}$). Alteration of rocks belonging to the former group, which tend to have higher FeO_T, lower SiO_2, and higher CaO contents, favors andradite precipitation and hence H_2 generation, as shown by the hotter-colored symbols for rocks with MgO contents <25 wt% in Figures S2a, S2b, and S2c, respectively.

The consequences of mixing among fluids of variable composition resulting from serpentinization are illustrated in the colored dots and curves in Figure S2. Specifically, colored dots in Figure S2a show results of a million calculations that simulate various extents of mixing (various colors in Figure S2a) of randomly selected fluids produced during alteration of mafic rocks (MgO <10 wt%) with those generated from reactions with rocks of similar or higher MgO contents. Colored curves in Figure S2b correspond to the distribution of colored dots in Figure S2a in a given compositional group. Due to orders-of-magnitude higher H_2-generation potentials, input from fluids equilibrated with rocks containing >35 wt% MgO dictates the concentration of H_2 in fluids from any heterogenous mafic-ultramafic body. As an example, to yield enough H_2 to offset O_2 production of 2 Tmole yr^{-1} (orange line in Figure S2b) at least 40% (dark-blue to violet dots and curves in Figure S2a and S2b, respectively) of groundwater in a total rock exposure of 18 million km^2 would need to interact with fluids from rocks with MgO content >35 wt%. Mixing

between fluids equilibrated with different rocks belonging to group 9 (MgO <10 wt%, colored curves in Figure S2b) results in a distribution that is different from that of end-member fluids (black curve for group 9 in Figure S2b). As an example, mixing of equal amounts (*e.g.*, 0.4 to 0.6 wt. fraction) of fluids belonging to extreme components in a non-normal and multimodal distribution (black curve for group 9 in Figure S2b) will result in a new distribution with a large number of values that are not in the original distribution depicting the end-member fluids. Mixing between fluids equilibrated with rocks with >35 wt% MgO content and those with MgO contents between 10 and 25 wt%, instead of <10 wt% shown in Figure S2, would still result in the same trends due to the much higher H_2-generation potentials of the Mg-rich rocks.

Note that the calculations described above simulate low-temperature alteration processes in continental and submarine aquifers. H_2 concentrations measured from basalt-hosted submarine vent fluids (which can attain values >1 *m*molal) and their ultramafic analogs (>10 *m*molal)[15] are less divergent than their low-temperature continental counterparts. Higher temperature water-rock reactions (>300 °C) occurring in continental geothermal aquifers and submarine hydrothermal systems can lead to the formation of other mineral assemblages. At higher temperatures, epidote which is a Fe(III)-bearing Ca and Al hydrous silicate mineral, is favored to form during hydrothermal alteration of basalts and results in the formation of H_2 (*e.g.*, ref. 16). In addition, further simulations that consider incorporation of ferric iron into chlorite, which has been observed in natural samples[3], albeit at lesser extents than those measured in serpentine[1,2], can refine

estimates on H_2 production during basalt and picritic rock alteration where chlorite formation can be significant.

As a summary, the H_2-generation potential of different Fe-bearing igneous rocks decreases with the MgO content of the rocks, as shown in Figures 1, 2, S1, and S2. As a consequence, alteration of these rocks would yield variable annual O_2-consumption potentials, as shown in Figures 4 and S3, which depict results of simulations assuming alteration rates of 10^{-6} and $10^{-6.5}$ km yr^{-1}, respectively. Accounting for fluids with variable starting redox states (*i.e.*, equilibrated at various atmospheric contents of O_2) does not considerably affect our models at the water-to-rock ratios considered in this work (<100), as shown in Figure S4.

Supplementary Figures

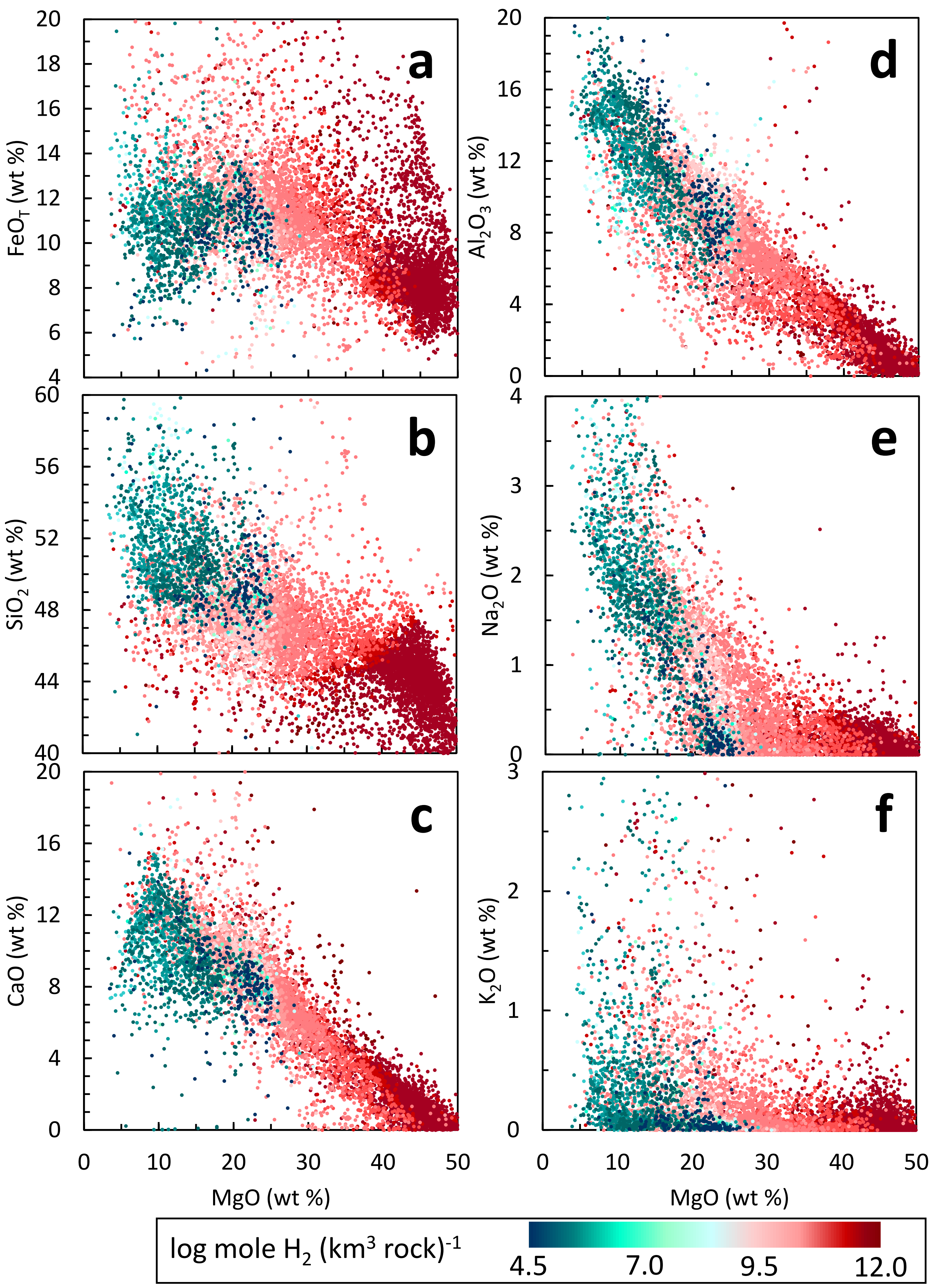

a
b
c
d
e
f
FeO_T (wt %)
SiO_2 (wt %)
CaO (wt %)
Al_2O_3 (wt %)
Na_2O (wt %)
K_2O (wt %)
MgO (wt %)
MgO (wt %)
log mole H_2 (km^3 rock)$^{-1}$
4.5
7.0
9.5
12.0

Figure S1. **Relationship between H_2 generation and rock compositions.** Influences of the major oxide compositions of rocks on calculated amounts of H_2 generated during hydrous alteration. The FeO (a), SiO_2 (b), CaO (c), Al_2O_3 (d), Na_2O (e), and K_2O (f) contents of compositions used in the simulations are plotted against their MgO contents, while symbol color represents amount of H_2 produced (log mole H_2 (km^3 rock)$^{-1}$).

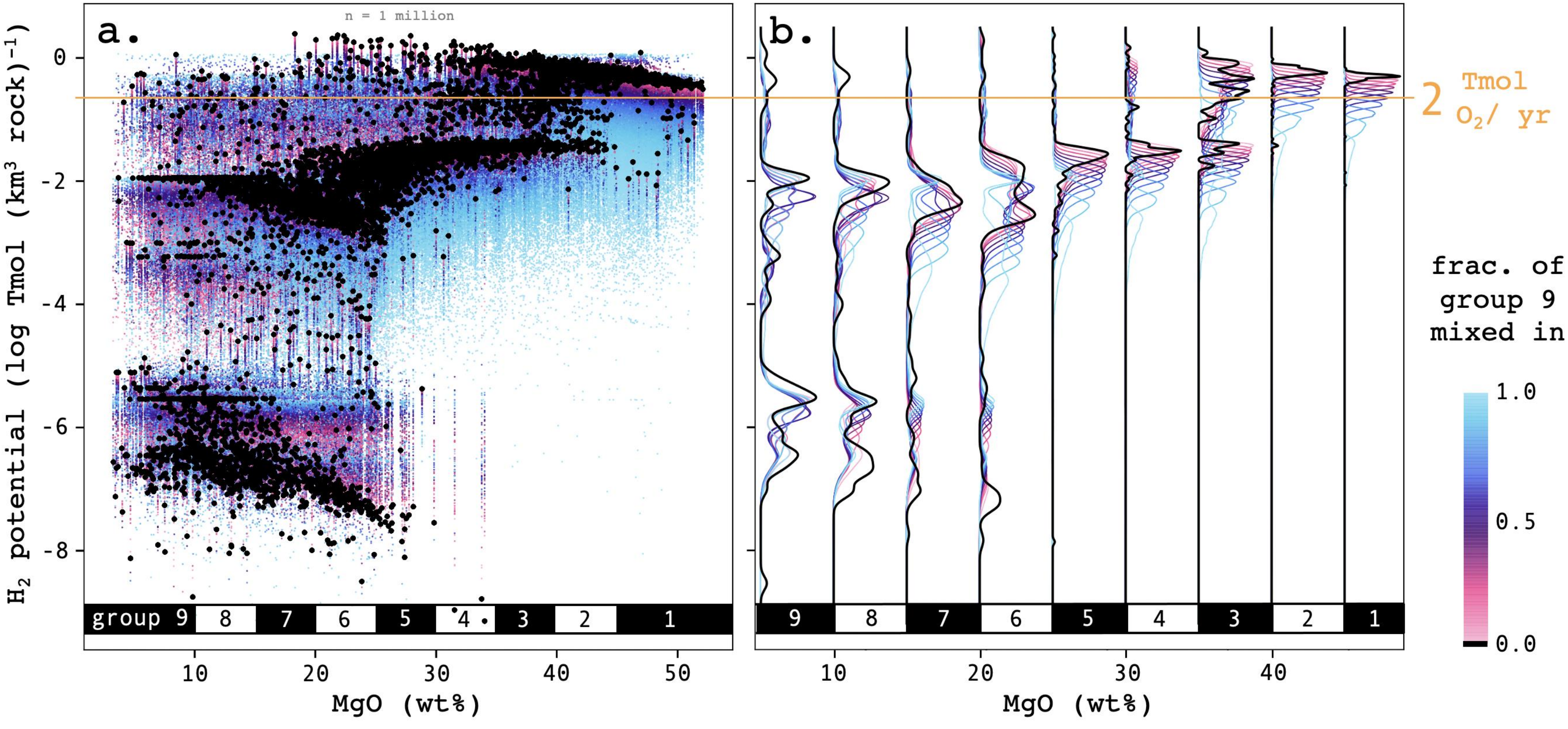
a.
b.
n = 1 million
H2 potential (log Tmol (km3 rock)-1)
0
-2
-4
-6
-8
group 9
8
7
6
5
4
3
2
1
10
20
30
40
50
MgO (wt%)
2 Tmol O2/ yr
frac. of group 9 mixed in
1.0
0.5
0.0

Figure S2. H_2-generation potential of rocks of variable compositions. (a) Similar to Figure 1a but y-axis (H_2-generation potential) in logarithmic values. Black dots correspond to grey dots in Figure 1a. In addition, colored dots indicate H_2 potentials of variable mixing of fluids equilibrated with mafic rocks (MgO <10 wt%) with those equilibrated with similar or more MgO-rich rocks. Results are shown for ~1,000,000 random combinations. Due to orders of magnitude higher H_2-generation potentials, input from fluids equilibrated with rocks containing >35 wt% MgO controls H_2 outgassing in a heterogenous mafic-ultramafic body. Colored symbols indicate mixing extent at 10% intervals. Black curves in (b) shows distribution of H_2-generation potentials of indicated compositional groups: (1) >45, (2) 40–45, (3) 35–40, (4) 30–35, (5), 25–30, (6), 20–25, (7) 15–20, (8) 10–15, and (9) <10 wt% MgO. Colored curves in (b) indicate distribution of H_2 potentials indicated by equivalent-colored symbols in (a) for mixing calculations. The orange line depicts H_2-generation potential required to offset O_2 production of 2 Tmole yr^{-1}, assuming alteration rate of 10^{-6} km yr^{-1} and total rock exposure of 18 million km^2 (*i.e.*, extent of oceanic plateaus, see discussions on seafloor serpentinization on the main text).

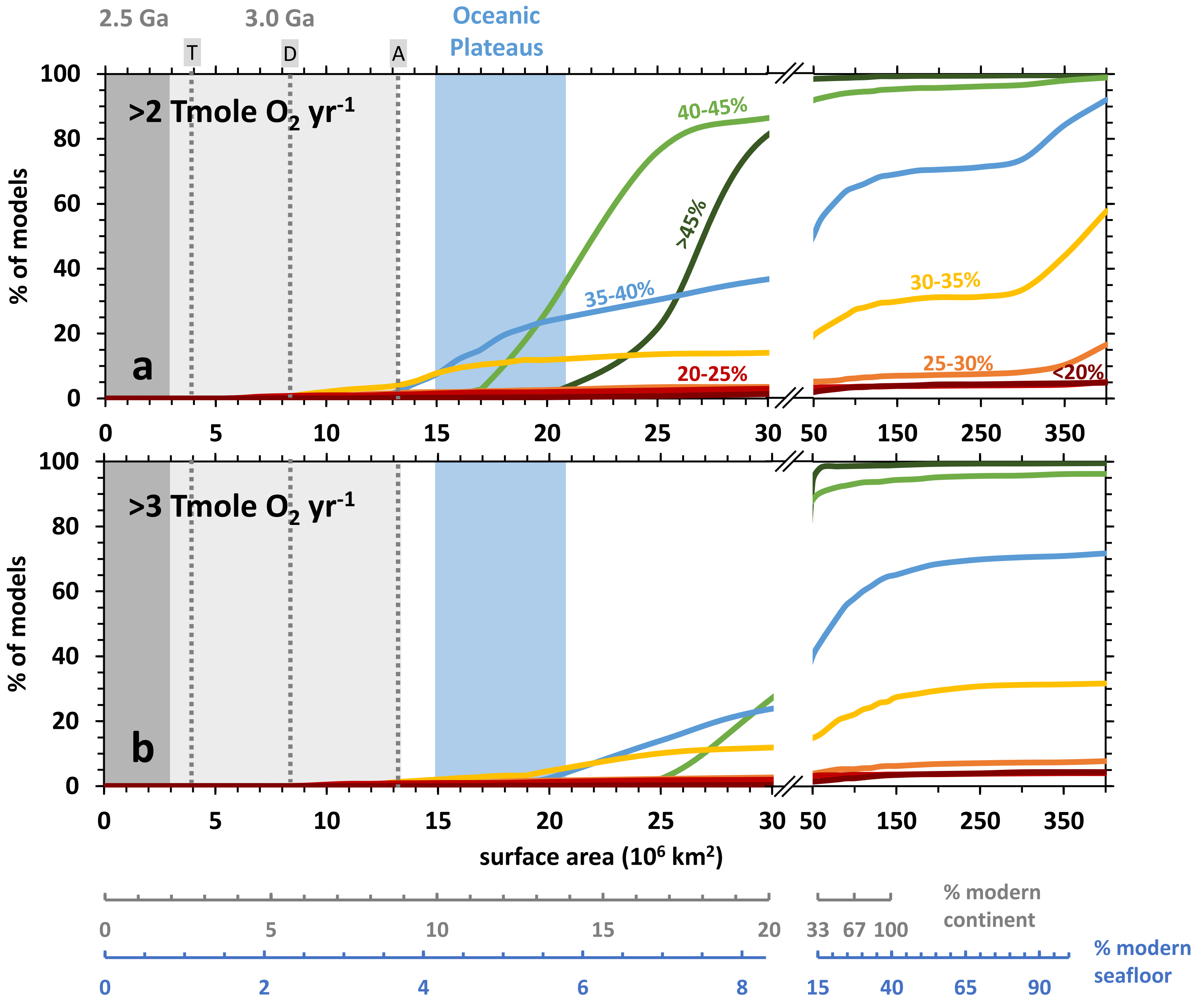
2.5 Ga
3.0 Ga
T
D
A
Oceanic Plateaus
% of models
>2 Tmole O_2 yr^{-1}
a
40-45%
>45%
35-40%
30-35%
25-30%
20-25%
<20%
>3 Tmole O_2 yr^{-1}
b
surface area (10^6 km^2)
% modern continent
% modern seafloor

Figure S3. O_2 consumption assuming alteration rate of $10^{-6.5}$ km yr^{-1}. Potentials for consuming more than 2 (a) or 3 (b) Tmole O_2 yr^{-1} as functions of the extent of komatiites exposed in continents (in km^2). Results are from models assuming serpentinization rate = $10^{-6.5}$ km yr^{-1}. Rightmost and leftmost shaded areas depict estimated ranges in the amount of komatiite exposed in continents 3.0 and 2.5 Ga ago, respectively. Vertical dashed grey lines depict maximum extent of ultramafic or komatiite exposures at 3.0 Ga based on compositional estimates of Greber et al.[17] and continental growth models of (A) Armstrong[18], (D) Dhuime et al.[19], and (T) Taylor and McLennan[20]. Other continental growth models[21,22] yields much lower maximum ultramafic extents. Blue shaded area represents extent of oceanic plateaus at the present-day (~5±1%, 18±3 million km^2 of the seafloor)[23]. Curves of various colors depict results of models simulating rocks belonging to different compositional groups as indicated by the MgO content.

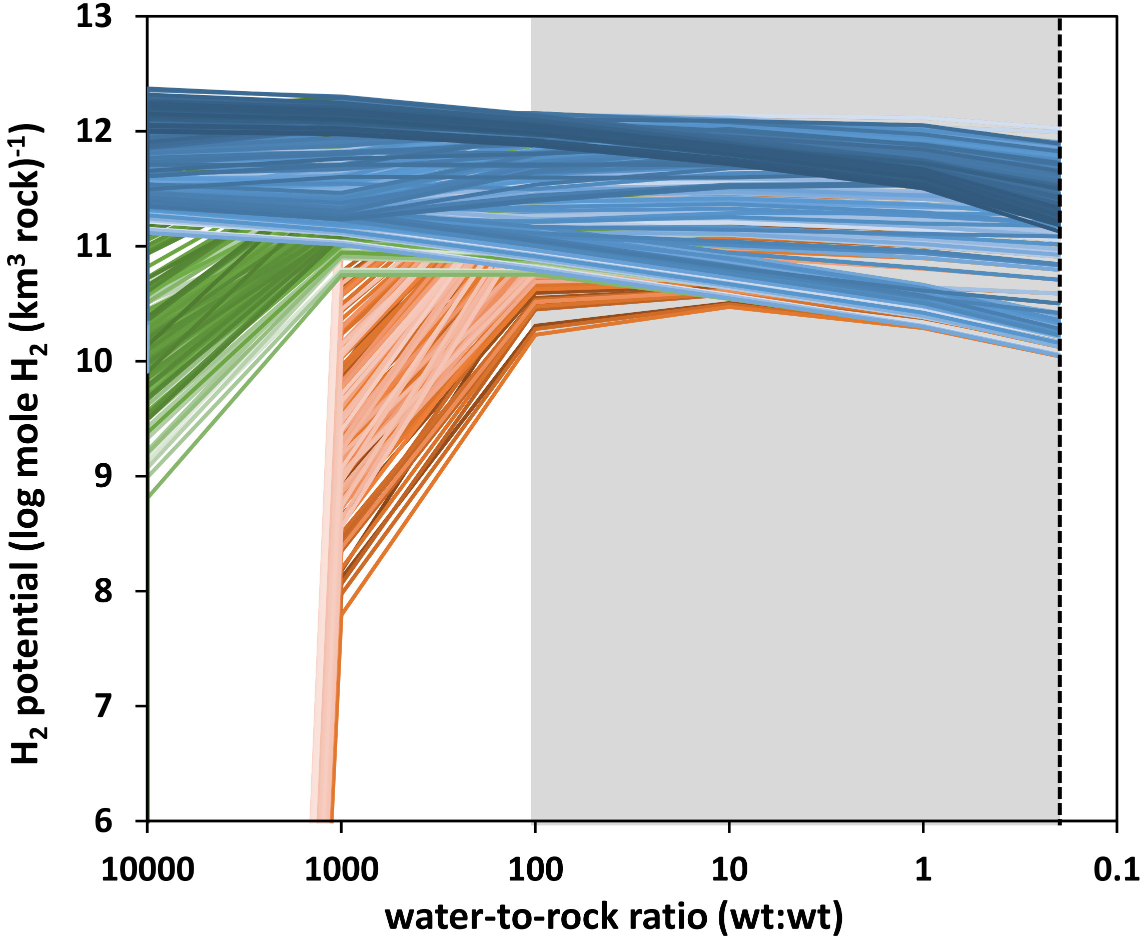

H2 potential (log mole H2 (km3 rock)-1)
13
12
11
10
9
8
7
6
10000
1000
100
10
1
0.1
water-to-rock ratio (wt:wt)

Figure S4. **H_2-generation potentials and water-rock ratios.** Calculated amounts of H_2 generated during hydrous alteration of a subset of the most MgO-rich samples from the GEOROC komatiite precompiled list (N = 250) at various degrees of water-to-rock ratios (x-axis). Left and right sides of the figure depict initial (high water-to-rock ratio or water-dominated systems) and advanced (low water-to-rock ratio or rock dominated systems) stages in the rock alteration progress, respectively. Colors depict results of simulations using various fluids with different starting dissolved O_2 in equilibrium with atmospheres containing $10^{-0.7}$ bar (modern levels, orange curves), 10^{-2} bar (green curves), and 10^{-3} bar (blue curves) of $O_{2(g)}$. Vertical dashed line indicates the lowest water-to-rock ratio (0.2) simulated. Grey field indicates conditions where water-to-rock ratio is <100, which are used in calculations of global H_2 production rates.

References

1. Andreani, M., Muñoz, M., Marcaillou, C. & Delacour, A. µXANES study of iron redox state in serpentine during oceanic serpentinization. *Lithos* **178**, 70–83 (2013).
2. Mayhew, L. E. & Ellison, E. T. A synthesis and meta-analysis of the Fe chemistry of serpentinites and serpentine minerals. *Philos. Trans. R. Soc. Math. Phys. Eng. Sci.* **378**, 20180420 (2020).
3. Dyar, M. D., Lowe, E. W., Guidotti, C. V. & Delaney, J. S. Fe3+ and Fe2+ partitioning among silicates in metapelites: A synchrotron micro-XANES study. *Am. Mineral.* **87**, 514–522 (2002).
4. Leong, J. A. M. & Shock, E. L. Thermodynamic constraints on the geochemistry of low-temperature, continental, serpentinization-generated fluids. *Am. J. Sci.* **320**, 185–235 (2020).
5. Tutolo, B. M., Seyfried, W. E. & Tosca, N. J. A seawater throttle on H2 production in Precambrian serpentinizing systems. *Proc. Natl. Acad. Sci.* **117**, 14756–14763 (2020).
6. Arndt, N. T., Lesher, C. M. & Barnes, S. J. Mineralogy. in *Komatiite* 98–129 (Cambridge University Press, 2008). doi:10.1017/CBO9780511535550.005.
7. Evans, B. W. The serpentinite multisystem revisited: Chrysotile is metastable. *Int. Geol. Rev.* **46**, 479–506 (2004).
8. de Caritat, P., Hutcheon, I. & Walshe, J. L. Chlorite Geothermometry: A Review. *Clays Clay Miner.* **41**, 219–239 (1993).
9. Barnes, I., O'Neil, J. R. & Trescases, J. J. Present day serpentinization in New Caledonia, Oman and Yugoslavia. *Geochim. Cosmochim. Acta* **42**, 144–145 (1978).
10. Sturchio, N. C., Abrajano, T. A., Murowchick, J. B. & Muehlenbachs, K. Serpentinization of the Acoje massif, Zambales ophiolite, Philippines: hydrogen and oxygen isotope geochemistry. *Tectonophysics* **168**, 101–107 (1989).
11. Kyser, T. K., O'Hanley, D. S. & Wicks, F. J. The origin of fluids associated with serpentinization; evidence from stable-isotope compositions. *Can. Mineral.* **37**, 223–237 (1999).
12. Leong, J. A. M. *et al.* Theoretical predictions versus environmental observations on serpentinization fluids: Lessons from the Samail ophiolite in Oman. *J. Geophys. Res. Solid Earth* **126**, e2020JB020756 (2021).
13. Paukert Vankeuren, A. N., Matter, J. M., Stute, M. & Kelemen, P. B. Multitracer determination of apparent groundwater ages in peridotite aquifers within the Samail ophiolite, Sultanate of Oman. *Earth Planet. Sci. Lett.* **516**, 37–48 (2019).
14. Plümper, O., Beinlich, A., Bach, W., Janots, E. & Austrheim, H. Garnets within geode-like serpentinite veins: Implications for element transport, hydrogen production and life-supporting environment formation. *Geochim. Cosmochim. Acta* **141**, 454–471 (2014).
15. Humphris, S. E. & Klein, F. Progress in deciphering the controls on the geochemistry of fluids in seafloor hydrothermal systems. *Annu. Rev. Mar. Sci.* **10**, 315–343 (2018).
16. Seyfried, W. E., Ding, K. & Berndt, M. E. Phase equilibria constraints on the chemistry of hot spring fluids at mid-ocean ridges. *Geochim. Cosmochim. Acta* **55**, 3559–3580 (1991).

17. Greber, N. D. *et al.* Titanium isotopic evidence for felsic crust and plate tectonics 3.5 billion years ago. *Science* **357**, 1271–1274 (2017).
18. Armstrong, R. L. Radiogenic isotopes: The case for crustal recycling on a near-steady-state no-continental-growth Earth. *Philos. Trans. R. Soc. Lond. Ser. Math. Phys. Sci.* **301**, 443–472 (1981).
19. Dhuime, B., Hawkesworth, C. J., Cawood, P. A. & Storey, C. D. A change in the geodynamics of continental growth 3 billion Years Ago. *Science* **335**, 1334–1336 (2012).
20. Taylor, S. R. & McLennan, S. M. *The continental crust: its composition and evolution: an examination of the geochem. record preserved in sedimentary rocks*. (Blackwell, 1985).
21. Allègre, C. J. & Rousseau, D. The growth of the continent through geological time studied by Nd isotope analysis of shales. *Earth Planet. Sci. Lett.* **67**, 19–34 (1984).
22. Condie, K. C. & Aster, R. C. Episodic zircon age spectra of orogenic granitoids: The supercontinent connection and continental growth. *Precambrian Res.* **180**, 227–236 (2010).
23. Harris, P. T., Macmillan-Lawler, M., Rupp, J. & Baker, E. K. Geomorphology of the oceans. *Mar. Geol.* **352**, 4–24 (2014).
